# Interaction studies of carbon nanomaterials and plasma activated carbon nanomaterials solution with telomere binding protein

**DOI:** 10.1038/s41598-017-02690-4

**Published:** 2017-06-01

**Authors:** Pankaj Attri, Jitender Gaur, Sooho Choi, Minsup Kim, Rohit Bhatia, Naresh Kumar, Ji Hoon Park, Art. E. Cho, Eun Ha Choi, Weontae Lee

**Affiliations:** 1 Plasma Bioscience Research Center/Department of Electrical and Biological Physics, Kwangwoon University, Seoul, 139–701 Korea; 2 Division of Sustainable Technology, Rudraksh Proudhyogiki Sangathan, Delhi, India; 3 Department of Biochemistry, College of Life Science & Biotechnology, Yonsei University, Seoul, 120–749 Korea; 4 Department of Bioinformatics, Korea University, Sejong, 02841 Korea

## Abstract

Most cancer cells have telomerase activity because they can express the human telomerase reverse transcriptase (hTERT) gene. Therefore, the inhibition of the hTERT expression can play an important role in controlling cancer cell proliferation. Our current study aims to inhibit hTERT expression. For this, we synthesized graphene oxide (GO) and a functionalized multiwall carbon nanotube (f-MWCNT), latter treated them with cold atmospheric pressure plasma for further analysis of the hTERT expression. The inhibition of hTERT expression by GO, f-MWCNT, plasma activated GO solution (PGOS), and plasma activated f-MWCNT solution (PCNTS), was studied using two lung cancer cell lines, A549 and H460. The hTERT experimental results revealed that GO and PGOS sufficiently decreased the hTERT concentration, while f-MWCNT and PCNTS were unable to inhibit the hTERT concentration. Therefore, to understand the inhibition mechanism of hTERT, we studied the binding properties of GO and PGOS with telomere binding protein (AtTRB2). The interaction studies were carried out using circular dichroism, fluorescence, ^1^H-^15^N NMR spectroscopy, and size-exclusion chromatography (SEC) binding assay. We also used docking simulation to have an better understanding of the interactions between GO nanosheets and AtTRB2 protein. Our results may provide new insights that can benefit in biomedical treatments.

## Introduction

In all eukaryotes, telomeres are essential for genome stability, and any abnormalities in telomere functions can result in human aging and cancer^1^. Telomeres consist of repetitive sequences that can protect the degradation of genomes^2^. In normal somatic cells, the end-replication problem reduces the length of the telomeres with each cell division, resulting in senescence^3^. On the other hand, in tumor cells, the telomerase replaces the telomere repeats that are lost during replication. Telomerase consists of a reverse transcriptase (TERT), which transmits its own form of an RNA moiety template^4^. Therefore, telomerase activity not only helps to maintain the telomeres of proliferating cells but also those occupied in the route of cellular immortalization and oncogenesis^5^. A strong correlation exists between the presence of hTERT mRNA and telomerase activity because hTERT expression depends on the rate of transcription^4, 6^. However, a method used to inhibit telomerase activity involves stabilizing the G-quadruplex structure^7, 8^. Chen *et al*. showed that single walled carbon nanotubes (SWCNTs) can inhibit telomerase activity through the stabilization of i-motif structures^7^. They found that SWCNTs can induce the uncapping of telomere and remove the telomere-binding protein that results in the aberration of telomere and cell growth cessation.

For the last many years, nanocarbon materials have played an important role in the development of new or improved technologies in various areas such as biomedical materials, renewable energies, supercapacitors, solar cells etc.^9–13^. In recent years, nanocarbon materials such as carbon nanotubes (CNTs), graphene, and graphene oxide have become emerging novel nanomaterials for delivering a variety of therapeutic agents, bioimaging, and other biomedical applications^14–23^. To increase the utility of nanocarbon materials for biomedical application, it is important to understand the interaction between carbon materials and proteins. In response to this, Kenry *et al*. recently showed the molecular interaction of GO with human blood plasma proteins^24^. In another recent work, Shin *et al*. showed the sensitive detection of the intracellular RNA of human telomerase using GO^25^. Chatterjee *et al*., indicated that GO treatment regulated the genes related to the response to oxygen levels, the regulation of cell growth and apoptosis, and metabolic processes^26^. Conversely, it was also observed that GO is highly toxic when administered directly to the lungs of mice, causing severe and persistent lung injury^27^. And review by Zhang *et al*., reveals that GO treatment alters the impacts cell growth, metabolic processes, and induces apoptosis (at higher concentrations of GO treatment)^28^.

Nowadays, nonthermal atmospheric pressure plasma has been applied in various areas such as sterilization, wound healing, anticancer therapy, etc.^29–36^. Moreover, the combination of plasma and carbon materials for biological applications is in its beginning stage^37, 38^. In our previous study, we observed that Fibronection adsorption is enhanced on N_2_ plasma-treated graphene^39^. Also, in our early work, we showed that plasma treated GO can increase the efficiency of solar cells^11^. Additionally, in recent years, many biomedical applications have been demonstrated for plasma activated water, medium, buffers, and compounds^40–44^. Therefore, in the present study, our aim is to study the effect of nano carbon materials on inhibition of hTERT expression and its probable mechanism. Therefore, we have synthesized GO through the unzipping of carbon nano horns by an oxidation reaction under mild conditions, resulting in improved and uniform morphology GO. And also synthesized the functionalization of MWCNT through the cycloaddition of aromatic azides, which results in detangled f-MWCNT, as described in earlier work^12, 13^. Thereafter, both GO and f-MWCNT were dissolved in DI water and treated with plasma to generate the plasma activated GO solution (PGOS) and plasma activated f-MWCNT solution (PCNTS). Further, we used the GO, f-MWCNT, PGOS, and PCNTS treat two lung cancer cell lines, A549 and H460 and check their effect on hTERT expression. To understand the hTERT expression inhibition mechanism, we then studied the interaction properties of GO and PGOS with telomere binding protein, AtTRB2. AtTRB2 protein has an N-terminal Myb domain, that is homologous to other telomere binding proteins both in plants and animals^45–49^. Therefore, we studied the interaction of GO and PGOS with AtTRB2 protein as model protein, using circular dichroism, fluorescence, ^1^H-^15^N NMR, and size-exclusion chromatography (SEC) binding assay. We also used docking simulation to better understand the interaction between GO nanosheets and AtTRB2 protein. ^1^H-^15^N NMR and docking studies help to understand the role of specific amino acids that participate in binding.

## Methods and Materials

### Materials

The reagents used for the synthesis of GO sheets and MWCNT were supplied by Merck Germany. The carbon nano horns were provided by JK Impax, India. All chemicals and reagents were used without any further purification. Chemical reagents of AR grade were used for the experimental work. The concentration of H_2_O_2_, NO_2_^−^ and NO_3_^−^ were measured as per method provided in our previous work^50, 51^.

### Synthesis of graphene oxide by unzipping of carbon nanohorns (CNHs)

The CNHs were suspended in 98% Sulfuric acid (H_2_SO_4_) for a period of 1 h with slow stirring. These suspended CNHs were treated with 500 wt% potassium permanganate (KMnO_4_) dissolved in H_2_SO_4_. The addition of the acidic solution of KMnO_4_ to the CNH suspension was performed very slowly with all due precautions because the CNH suspension will usually catch fire if the solutions are mixed together too rapidly. The reaction mixture was continuously stirred and after 1 h it was slowly heated to 50 °C. After 2 h, the reaction mixture was cooled to room temperature followed by reaction with 3 wt% hydrogen peroxide (H_2_O_2_) to reduce the MnO_2_ to water soluble Mn^2+^. The product was highly soluble in the acidic solution; it was therefore centrifuged at 10,000 rpm for 10 min and the acid was carefully decanted. The product was then mixed with deionized (DI) water and again centrifuged for 10 min at 10,000 rpm; this washing was repeated until the pH of the water solution became neutral. Before using the product for film casting, the GO sheets were re-suspended in water and subjected to ultra-sonication for 20 min at 45 kHz. This was performed to further exfoliate the GO sheets, and homogenous suspension was achieved.

### Functionalization of Carbon Nanotubes

The functionalized MWCNT was synthesised according to the method described in our previous research^12, 13^. The precipitated product (*f*-MWCNT) was obtained by centrifugation with repeated washing with DI water and dried in an oven at 75 °C. Spectral data; IR (cm^−1^): 1733 (C=O), 1598 (C=C), 1435 (C-N), 1254 (C-O stretch), 1139 (C-O out of plane deformation), and 836 (C-H).

### Soft plasma jet details

Soft plasma jet consists of a needle-type powered electrode which is enclosed by a quartz tube with an inner diameter of 3 mm, an outer diameter of 5 mm, and a length of 9 mm. To perform this experiment, the distance between the powered electrode and the sample surface was set at 4 mm and nitrogen (N_2_) gas was used as the feeding gas with a flow rate of 2 l/m. The gas was injected into the needle of the plasma jet and was then extracted through a 1mm hole. The spectra of the soft jet emission are recorded using a HR4000CG-UV-NIR (Ocean Optics, FL, USA) over a wide wavelength range of 200–1100 nm, with a humidity of 40% inside the treatment chamber.

### Preparation of plasma activated GO solution and plasma activated f-MWCNT solution

To prepare the plasma activated GO solution (PGOS) and plasma activated f-MWCNT solution (PCNTS), we first prepared the GO and f-MWCNT as described above. We then prepared a 1 mg/ml stock solution of GO and f-MWCNT in DI water and ultra-sonicated the solution for 30 min at room temperature. Further, we treated the sonicated GO and f-MWCNT with a soft plasma jet using N_2_ as the feed gas for 5 min to generate the PGOS and PCNTS.

### Cell viability and hTERT concertation assay

Human lung cancer cell lines [A549 (American Type Culture Collection (ATCC) and H460 (Korean Cell Line Bank (KCLB)] were used to check the effects on cell viability and hTERT expression in the presence of GO, f-MWCNT, PGOS and PCNTS. Both cells were maintained in Dulbecco’s modified Eagle’s medium (DMEM; WEL GENE) supplemented with 10% fetal bovine serum, 1% non-essential amino acids, 1% glutamine, 1% penicillin (100 IU/ml) and streptomycin (100 mg/ml) (all from Hyclone, USA). All cultures were maintained at 37 °C, 95% relative humidity and 5% CO_2_. The cells allowed to grow in 75 cm^2^ tissue culture flasks until confluence were then sub-cultured for experimentation. Latter, 24 well plate contain 2 ~ 4 × 10^5^ cells/1 ml in complete media were exposed to 1 to 14 µg/ml concentration of GO, f-MWCNT, PGOS and PCNTS (without exposure (0) was included in each assay) than plates were transfer for 72 h incubation times. After incubation both exposed and non-exposed cells were subjected to measured hTERT concentration by using LSBio™ human TERT/Telomerase ELISA kit (LifeSpan BioScience, Inc), as well also performed the cell viability experiment using MTT assay.

### Construct design, protein expression and purification

The AtTRB2 cDNA was cloned into the pET15b vector (Novagen) including His-tag and a chaperon-like functional peptide (GRIFLQD). The specific cleavage sequence of the tobacco etch virus (TEV) protease, ENLYFQG, was induced in *E coli* BL21 (DE3) cells (Novagen) with 1 mM isopropyl-1-thio-β-D-thiogalactoside (IPTG) in optical density values of 0.6 at 600 nm. After 12 h of incubation, the cultured cells were harvested and the cell pellet was broken up in the lysis buffer, 10 mM HEPES (pH 7.0), and 100 mM NaCl using sonication. After centrifugation at 15,000 rpm, at 4 °C for 30 min, the collected inclusion bodies were solubilized in a binding buffer including 6 M Gdn-HCl at room temperature for 12 h. After resolubilization, the insoluble pellet was removed using centrifugation, and a soluble fraction was used for nickel affinity chromatography work. For the gradient refolding of target proteins, we followed the efficient on-column refolding scheme in Gel-filteration (AKTA™ prime) with HiTrap™ (GE Life Sciences). Refolded AtTRB2 was eluted in 10 mM HEPES (pH 7.0), 100 mM NaCl, and 500 mM imidazole. The His-tag was removed through the TEV protease, and pure AtTRB21-64 was isolated by size exclusion gel chromatography with a Hi Load Superdex 75 prep grade column (Pharmacia). For the preparation of the NMR samples, M9 minimal media and isotopes (^15^NH_4_Cl) were used during the cell culture.

### Measurements

The Raman spectra were measured at room temperature using a confocal Raman microscope (WITec, Alpha 300 R) with a 632.8 nm He-Ne laser. In order to obtain the Raman spectra for the GO sheets, the incident laser beam was focused onto the sample using a microscope objective (100×). The scattered light was collected by the same objective lens, then dispersed by a grating, and then detected by a charge-coupled-device array detector. The high-resolution XPS experiments were performed at the PLS-8A1 undulator (U7) beam line equipped with a variable-included-angle plane-grating monochromator. Scanning electron microscopy (SEM) was performed using JSM 7001F, JEOL, Tokyo, Japan.

### NMR spectroscopy

NMR experiments were performed in a mixture of a 90% H_2_O and 10% D_2_O NMR buffer, 10 mM HEPES (pH 7.0), 100 mM NaCl at 298 K on the Bruker DRX 500 Mhz equipped with CryoProbe. Sequential resonance assignment was executed by ^1^H -^15^N HSQC^47^. For NMR titration experiments, ^15^N labeled AtTRB2 was purified from M9 Media containing ^15^NH_4_Cl and the counter protein was purified from LB Media. ^1^H -^15^N HSQC spectra was used for titration experiments that were performed at carious molar ratios of ^15^N-labeled proteins with respect to GO. All collected spectra were processed and analyzed via XWIN NMR (Bruker Instruments, Karlsruhe, Germany), nmrPipe/nmrDraw (Biosym/Molecular simulation, Inc. San Diego, CA, USA) software, and the PINE-SPARKY program.

### Circular dichroism (CD) spectroscopy

Circular dichroism (CD) spectroscopic studies^52–55^ were performed using a J-815 spectrophotometer (Jasco, Japan) equipped with a Peltier system for controlling the temperature. (1S)-(+)-10-camphorsulfonic acid (Aldrich, Milwaukee, WI) was utilized for CD calibrations, exhibiting a molar extinction coefficient of 34.5 M/cm at 285 nm, and a molar ellipticity (θ) of 2.36 M/cm at 295 nm. The samples were pre-equilibrated at the desired temperature for 15 min and the scan speed was fixed for adaptive sampling (error F 0.01) with a response time of 1 s and 1 nm bandwidth. The secondary structure of AtTRB2 was monitored by using a 1.0 mm path length cuvette. The concentration for protein was 0.2 mg/ml in a buffer of 10 mM HEPES (pH 7.0) and 100 mM NaCl, with each spectrum being an average of six spectra. Each sample spectrum was obtained by subtracting the appropriate buffer without protein from the experimental proteins spectrum.

### Fluorescence spectroscopy

The fluorescence spectroscopy instrument used for measuring the fluorescence intensity in the present investigation is similar to that depicted in our earlier articles^52–55^. Steady-state fluorescence measurements were carried out using a Perkin Elmer LS 55 fluorescence spectrometer. The excitation wavelength was set at 290 nm for evaluating the contribution of the tryptophan residues to the overall fluorescence emission. The experiments were performed at 25 °C by using a 1 cm sealed cell. Both the excitation and emission slit width were set at 5 nm, and correction for the background signal was performed. The concentration for protein was 0.5 mg/ml in a buffer of 10 mM HEPES (pH 7.0) and 100 mM NaCl, with each spectrum being an average of six spectra.

### Size exclusion chromatography

Purified AtTRB2 samples were loaded onto a HiLoadTM 16/60 superdexTM75 (GE Life Science) gel filteration (AKTA™ prime) column equilibrated with the same final protein buffer [10 mM HEPES (pH 7.0) and 100 mM NaCl]. The size exclusion chromatography result was monitored by absorbance at 280 nm. The molecular weight of the purified protein was determined by reference to the standard proteins of albumin (66 kDa), carbonic anhydrase (19 kDa), and aprotinin (6.5 kDa), as shown in Fig. S1a. The molecular weight of AtTRB2 was calculated by the equation (log Y = −1.2483x + 6.2868, R^2^ = 0.9943, x = elution volume), as shown in Fig. S1b.

## Result and Discussion

### Synthesis and characterization of GO and f-MWCNT

**Figure 1:**
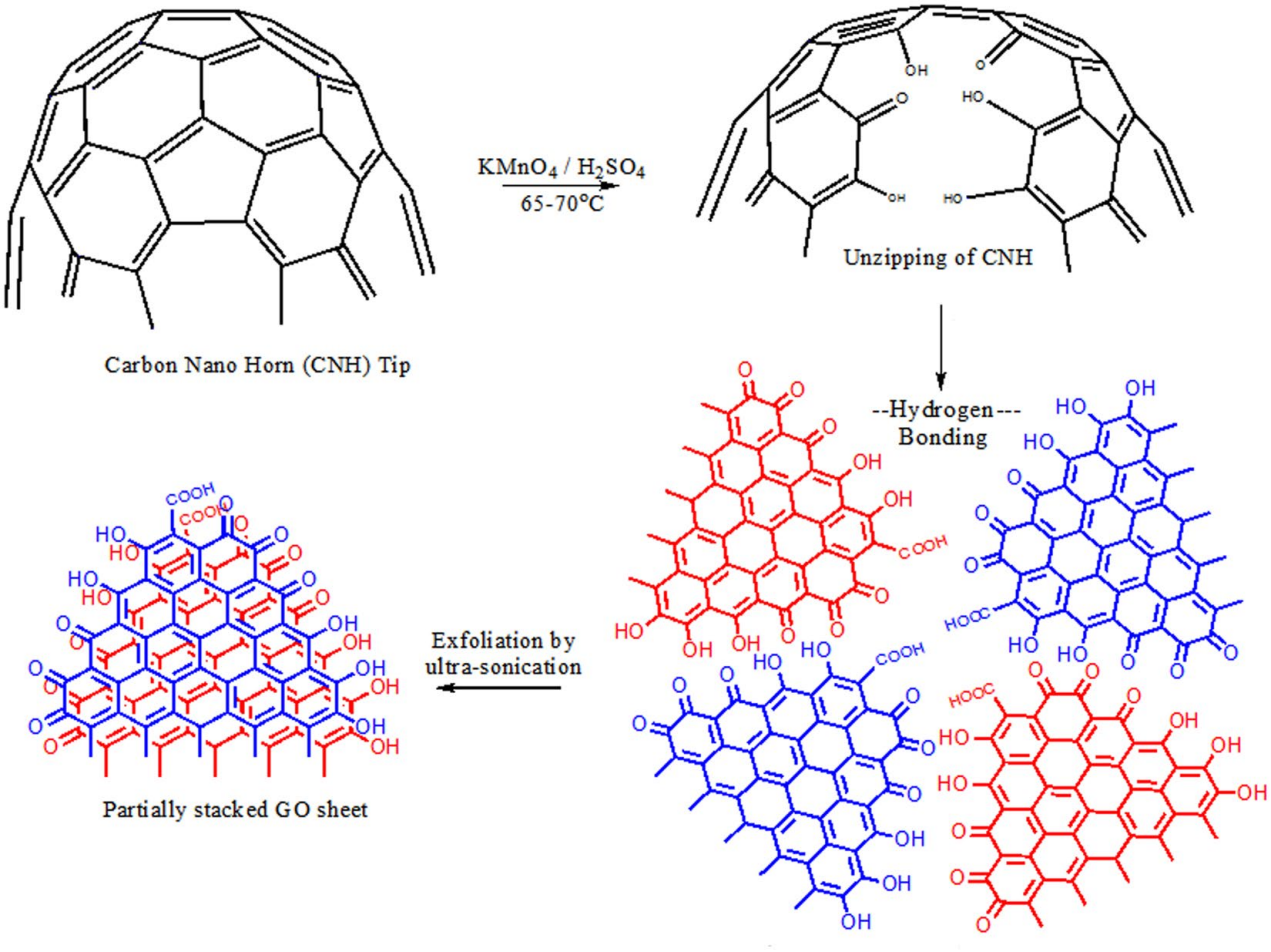
Schematic representation of the process of unzipping of CNHs into homogenous GO sheets with uniform morphology and their further exfoliation by ultrasonication to thin GO sheets.

CNHs were unzipped into highly ordered and water soluble thin GO sheets by a simple and innovative process as shown in Fig. 1. The process involved the treatment of CNHs with acidic KMnO_4_ under mild conditions to produce a highly water soluble dark brown product followed by extraction of GO and further exfoliation by ultra-sonication. The steep curvature at the tips of the CNHs have been shown to have 5 five–membered rings, which tend to break the extensive conjugation of electron density due to the angle strains in the five-membered rings^56–62^. This provides several isolated double bonds in the CNHs. These isolated double bonds can readily be oxidized by the KMnO_4_^63–65^. This feature of the readily available attack site for KMnO_4_ is not available in highly conjugated materials such as graphite and CNTs, and hence the need for a defect arises to initiate the reaction. This requires use of stronger and explosive oxidative materials such as sodium nitrate, perchlorates etc. to initiate the reaction^66–68^. The action of KMnO_4_ unzips the CNHs to produce GO sheets with a partial circular discs morphology. These discs consist of a number of GO sheets due to their extensive bonding through van der Waals stacking and hydrogen bonding involving the newly derived oxygen functional groups such as hydroxyl, carbonyl, epoxy, and carboxylic acid on the lateral edges of the graphene sheets. These clustered GO sheets with uniform morphology were exfoliated by ultrasonication to produce large scale thin GO nano sheets.

The chemical characterization of the above synthesized thin GO sheets was performed using X-ray photoelectron spectroscopy (XPS) and confocal Raman spectroscopy. Carbon 1 s XPS spectra shows the peak of the C–O, C=O, and COO functional groups in GO at 286, 287, and 289 eV, respectively, as shown in Fig. S2a. The most intense peak is at 284.6 eV, indicating the majority of C–C bonds in the GO sheets, signifying the large size of these sheets in the GO^68, 69^. Further, relative analysis of these signals shows that the alcoholic functional groups are dominant over the carbonyl and carboxylic functional groups. This is due to the mild oxidation conditions employed for the unzipping reaction. The Raman spectra (Fig. S2b) of the thin GO sheets (blue) show two bands at 1350 and 1607 cm^−1^, respectively, which correspond to the G (graphitic) and D (disordered) bands of the thin GO sheets. The presence of the G band indicates graphene characteristics in the thin GO sheets, representing the E_2g_ mode, i.e. in-plane bond stretching motion of pairs of sp^2^ hybridized atoms. Similarly, the D band at 1350 cm^−1^ has been attributed to the breathing modes of sp^2^ rings with non-planar functional groups or generally the previously mentioned defects^70, 71^. For thin GO sheets, the ratio of intensities of the D and G bands is greater than 1; i.e. I_D_/I_G_ > 1, which is characteristic for the oxidized edges of graphene developed by unzipping of CNHs into GO sheets.

The morphology of the products involved in the unzipping reaction was observed by SEM imaging. Figure S2c shows the SEM images of thin GO sheets bonded by hydrogen bonding and van der Waals bonding. This homogenous morphology of GO may be attributed to the availability of the identical sites in the structures of the CNHs. The corresponding electron diffraction pattern of the thin GO sheet is shown in Fig. S2d. The 6-fold symmetry in the diffraction pattern is consistent with the hexagonal structure generally present in the GO and labeled with Miller-Bravais indices^72^.

**Figure 2:**
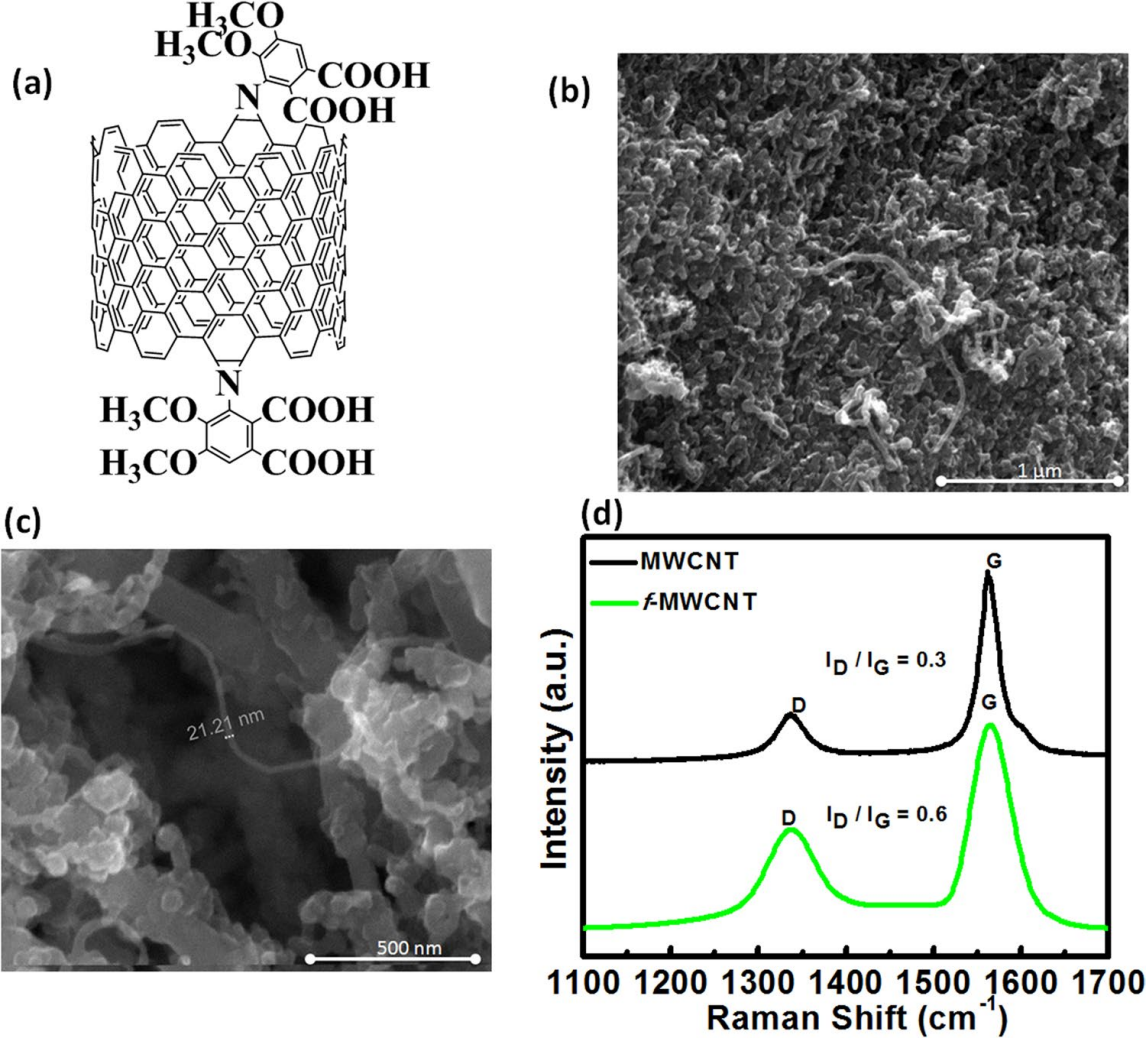
(**a**) Representation of the f-MWCNT structure; (**b**) SEM image of f-MWCNT from 1 µm; (**c**) SEM image of f-MWCNT from 500 nm, reveals the diameter of f-MWCNT; (**d**) Raman spectra, MWCNT (black) and f-MWCNT (green).

The synthesis of functionalized MWCNT was described in our early studies^12, 13^, and structure of the f-MWCNT is shown in Fig. 2a. It was shown that the 1,3-dipolar [3+2] cycloaddition of azides to the MWCNT sidewalls with thermal extrusion of N_2_ from the triazoline intermediate results in aziridino f-MWCNT. Through this method, we can protect the CNT structure from physical damage or breakage while retaining its high conductivity^73^. The diameter of f-MWCNT is found to be ~21 nm through SEM analysis, as shown in Fig. 2b and c. To identify the features that change through azide functionalization on MWCNT, we used confocal Raman spectroscopy, as shown in Fig. 2d. The Raman data show that the intensity ratio (I_D_/I_G_) of the D and G bands for Pristine MWCNTs was ~0.30, but after the formation of the f-MWCNT, the intensity ratio (I_D_/I_G_) increases to 0.60, demonstrating the presence of the sp^3^ carbon network after functionalization^74^; after functionalization, due to insertion reaction, a large amount of the sp^2^ carbon is converted to sp^3^, leading to an increment in the D band intensity.

### Reactive oxygen and nitrogen species (RONS) generated by soft plasma jet and structural analysis of GO and f-MWCNT after the plasma treatment

We analyzed the RONS generated from the soft plasma jet using N_2_ feeding gas inside a nano carbon material solution (PGOS and PCNTS). Optical emission spectra of the soft plasma jet show strong emission lines from a molecular NO β, γ system, and N_2_ second-positive system (C^3^П_u_–B^3^П_g_), as explained in our previous work^75^. We analyzed the concentration of H_2_O_2_, NO_2_^−^, and NO_3_^−^ in the DI water solution using chemical analysis and observed that the concentration of H_2_O_2_, NO_2_^−^, and NO_3_^−^ after 5 min treatment was 40 µM, 90 µM, and 130 µM, respectively.

We then used Raman analysis to determine whether or not structural changes had occurred in the GO and f-MWCNT after the plasma treatment. No change was observed in the Raman peak of both GO and f-MWCNT after the plasma treatment (data not shown); hence, both nanocarbon materials showed no change in the structure.

### Inhibition of hTERT expression in lung cancer cells after treatment with carbon nano-materials with or without plasma treatment

**Figure 3:**
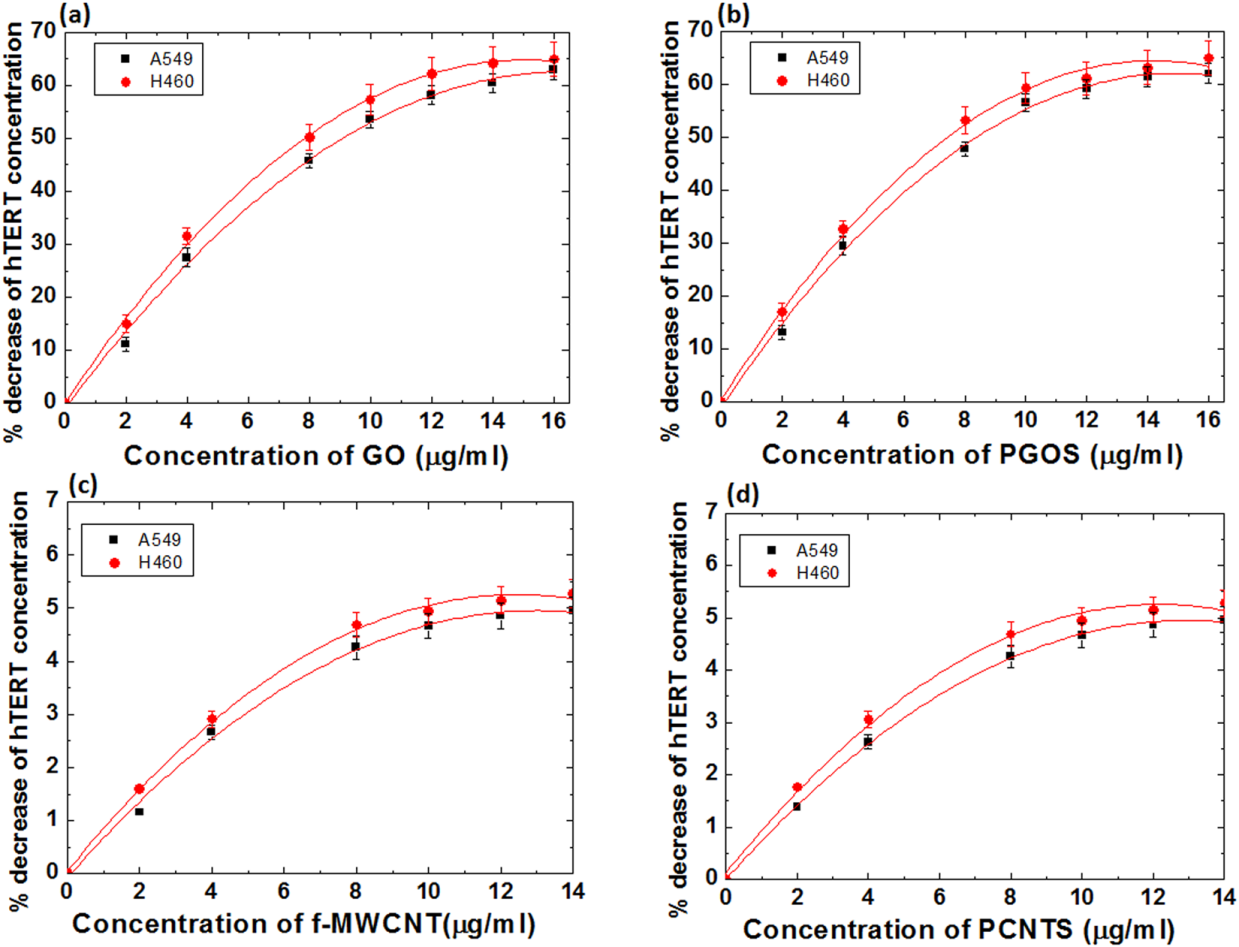
Percentage of decrease in hTERT concentration after (**a**) GO; (**b**)PGOS; (**c**)f-MWCNT and (**d**) PCNTS treatments on the A549 and H460 cancer cells.

In order to investigate the inhibition efficiency of the GO, f-MWCNT, PGOS, and PCNTS, we studied two lung cancer cells, A549 and H460. The trend of the use of GO and f-MWCNT for drug delivery and other bio-applications has recently increased^76, 77^. A549 and H460 lung cancer cells were cultured in the presence of GO, f-MWCNT, PGOS, and PCNTS at concentrations ranging from 1 to 14 μg/ml. After 3 days, the cells were lysed and hTERT expression was assessed. Figure 3 shows the clear concentration-dependent loss of hTERT for the GO and PGOS treatments, but this trend differs for the f-MWCNT and PCNTS treatments. hTERT expression inhibits in H460 cancer cells more than A549 cancer cells. In the present study, we observed that PGOS and GO show no significant difference in the inhibition of hTERT concentration for both cancer cells. Similar trends were also observed for f-MWCNT and PCNTS on both lung cancer cells. The f-MWNTs and PCNTS showed no significant inhibition of hTERT concentration, as seen in Fig. 3, due to the larger diameter of MWNTs (as described above). The treatment of two lung cancer cells in the presence of PGOS and PCNTS reveals that the RONS generated in both solutions do not significantly contribute to the decrease the hTERT concentration. The results of the f-MWCNT on hTERT concentration correlates with earlier work, where the authors found that MWCNT-COOH has no action on telomerase activity using K562 and HeLa cells^7^. The authors of ref. 7 also demonstrated that SWNT-COOH can induce telomere uncapping and the removal of telomere-binding protein from telomere, which results in telomere aberration and inhibition of telomerase activity^7^.

Recent study shows that GO has a tendency to interact with proteins^24^. In our present work, the one main reason for the decrease of hTERT concentration might be the interaction of the GO with telomerase binding protein and the later removal from telomere. Therefore, to understand the interaction of GO and PGOS with the telomerase binding protein, we used AtTRB2 as the model telomerase binding protein. Because AtTRB2 is a member of the Single-Myb-Histone-like family in Arabidopsis thaliana, they have an N-terminal Myb domain that is responsible for DNA binding^47^. Therefore, this interaction study provides information about the structural changes of AtTRB2 protein after binding and suggests the amino acid that participated in binding.

### Analysis of conformational change in AtTRB2 protein after exposure of GO and PGOS using circular dichroism spectroscopy

One of the crucial processes of the proteins and GO nanosheets interaction is the adsorption of protein on the nanomaterial surface. During this process, some proteins will retain their native-like structure or undergo conformational changes (change in secondary or tertiary structures) upon surface binding with GO nanosheets. In most of the nanomaterial-protein interactions studies, a certain degree of conformational change is reported to occur in the protein after binding^24^. Additionally, carbon nanomaterial surface binds at a specific orientation to the proteins. The conformational changes will therefore be influenced by the surface chemistry of the nanomaterials. These conformational changes result in a change in the functionality of proteins, loss of biological activity, and induce aggregation of proteins, which effects the interaction of these proteins with other cellular components. Therefore, it is important to understand the nature of the conformational changes that occurred in the telomere binding protein AtTRB2 upon interaction with the GO or PGOS nanosheets. We used CD spectroscopy to understand the change in the secondary structure of the telomere binding protein AtTRB2 upon interaction with the GO or PGOS nanosheets.

**Figure 4:**
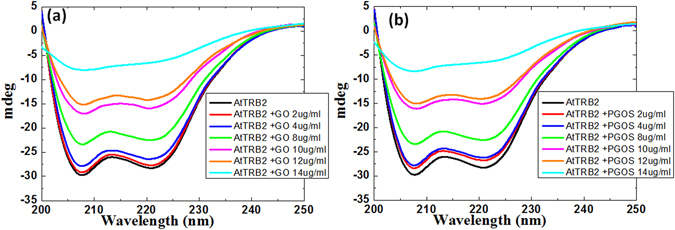
Secondary structure analysis using CD spectroscopy of AtTRB2 protein after treatment with (**a**) GO at different concentration and (**b**) at different concentration of PGOS.

The CD spectra from 200 to 250 nm are utilized to quantify the discrepancy in the secondary structure of the AtTRB2 protein due to the molecular interactions between protein and GO or PGOS nanosheets. Here, at the fixed concentration of AtTRB2 protein, we added the GO or PGOS nanosheets concentrations of 2, 4, 8, 10, 12, and 14 µg/ml. The CD spectra of the protein in the presence of the GO or PGOS nanosheets were then recorded as shown in Fig. 4. The secondary structure of AtTRB2 is predominantly α–helical and their CD spectra exhibited two distinct negative bands at 208 and 222 nm. The secondary structures of AtTRB2 that are stabilized in the presence of GO and PGOS nanosheets can be assessed by evaluating the ellipticity of the CD spectra at 208 and 222 nm. Figure 4 shows a progressive decrease in the ellipticity of the CD spectra of protein with the increasing concentrations of GO nanosheets. This result indicates the occurrence of strong molecular interactions between the AtTRB2 protein and GO nanosheets. Clearly, the CD spectra exhibited the greatest decrease in its ellipticity in the presence of the 14 µg/ml of GO nanosheet; similarly, for the PGOS nanosheet, the protein displayed the greatest decrease in its ellipticity. Although the intensities of the two characteristic bands at 208 and 222 nm were reduced by up to 12 µg/ml of GO or PGOS nanosheet concentration, these negative bands were still apparent with their α-helical structures. This can be explained as the ability of AtTRB2 protein to preserve its native conformational stability by maintaining its overall secondary structure upon the interactions with GO or PGOS nanosheets up to 12 µg/ml, while at a higher concentration of 14 µg/ml, its conformation decreases. Moreover, if we examine the change in ellipticity between the GO and PGOS binding with AtTRB2 protein, no significant difference is observed in both bindings, and they show the same level of structural changes, as shown in Figs S3 and S4. This result supports our decrease of hTERT concentration, where the inhibition efficiencies of GO and PGOS do not significantly differ to each other. For further detailed understanding of the quenching efficiency and binding ability of the GO and PGOS, we used steady state fluorescence spectroscopy and a size-exclusion chromatography (SEC) binding assay, respectively.

### Fluorescence quenching assay and size-exclusion chromatography (SEC) binding assay

**Figure 5:**
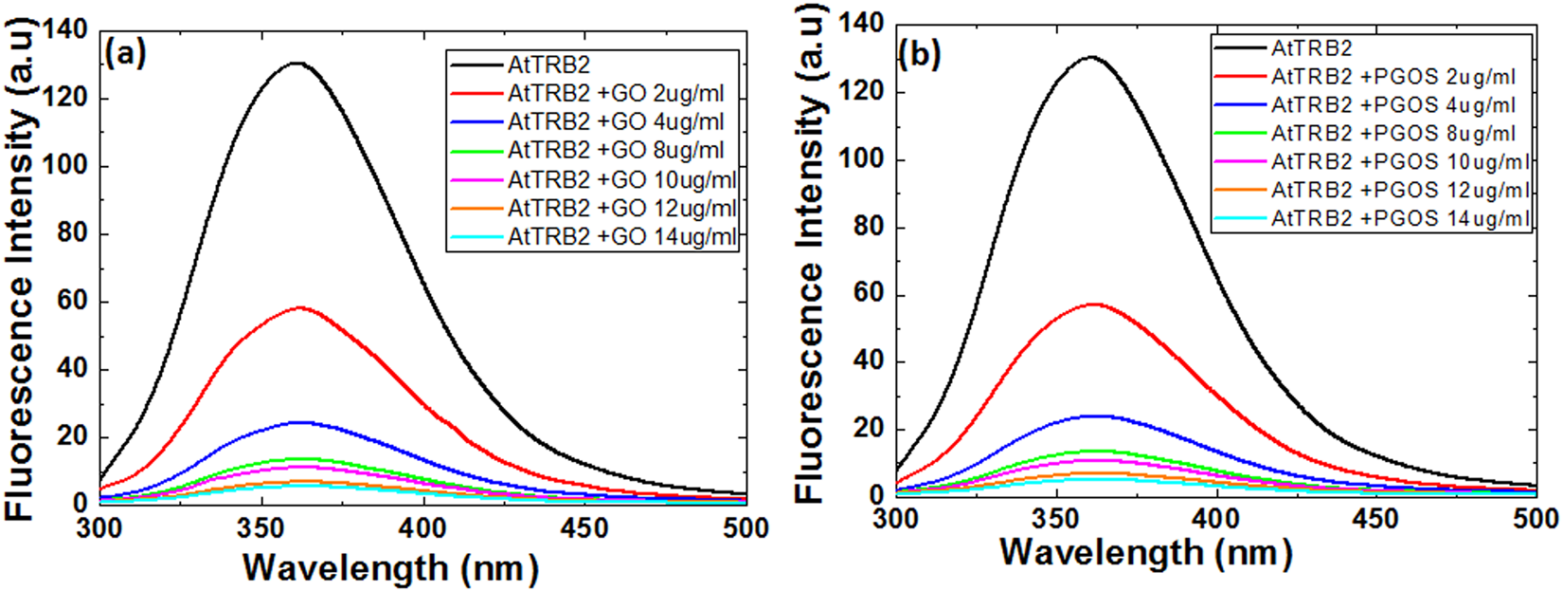
Quenching studies of AtTRB2 protein after treatment with (**a**) GO at different concentration and (**b**) at different concentration of PGOS using fluorescence spectroscopy.

Proteins consist of distinctive amino acids. The intrinsic protein fluorescence is due to the aromatic amino acids, generally tryptophan, tyrosine, and phenylalanine. Low quantum yield of intrinsic fluorescence is observed for phenylalanine, while the emission by tyrosine is often quenched in native proteins. These intrinsic fluorescent probes are very sensitive to the nature of their microenvironment, such as protein conformational transitions, ligand binding, subunit association, and denaturation, all of which affect the local environment surrounding the indole ring that results in shifts in emission spectrum or quenching. Studies have shown that the GO fluorescence quenching capability depends on its interaction with biomolecules^78^. Therefore, the discrepancy in the fluorescence emission intensity of telomere binding protein (AtTRB2) helps in the understanding of the change in conformation after the binding with GO. Our experimental data showed that the maximum emission intensity I_max_ of AtTRB2 protein gradually decreased as concentrations of GO increased, as illustrated in Fig. 5a. The quenching effect of chromophore residues in the AtTRB2 due to the presence of GO suggests the occurrence of direct interactions between GO and the chromophore residues of the AtTRB2 protein. The fluorescence quenching increases as the concentration of GO increases, showing that the amount of tryptophan and tyrosine residues of the bound AtTRB2 is accessible to the GO surface and is due to the change in conformation of AtTRB2 as a result of the binding of GO. We observed a slight red shift in λ_max_ values with the addition of GO. The I_max_ value for AtTRB2 protein without GO addition was 361 nm; with the addition of 2 µg/ml of GO, the I_max_ value was 361.5 nm. Similarly, with a further increase in GO concentrations of 4, 8, 10, 12, and 14 µg/ml, the I_max_ values were 362, 363, 363.5, 364, and 364.2 nm, respectively (Fig. 5b). This shows that with the increase in concentration of GO, the wavelength at the I_max_ value for the AtTRB2 protein is slightly red shifted compared to the control. The red shift in the wavelength shows that the protein structure is more hydrophilic after the addition of GO than before GO treatment, revealing that structure changes occur in the AtTRB2 protein.

Subsequently, the interaction of PGOS with AtTRB2 as a function of PGOS concentration results in a similar trend of quenching with a slight red shift in wavelength at maximum intensity (I_max_), as illustrated in Fig. 5b. With the additions of 2, 4, 8, 10, 12, and 14 µg/ml of PGOS, the I_max_ values were 361.5, 362.2, 362.3, 362.3, 362.4, and 362.5 nm, respectively, revealing that the red sift in the wavelength is significant for the low concentration of the PGOS addition, but is almost constant for the higher concentration of the PGOS addition to AtTRB2 (Fig. 5b). The apparent quenching effect with the addition of different concentrations of GO suggests the occurrence of direct interactions between GO and the chromophore residues of the AtTRB2 protein. In reality, decreases in the emission intensity of the protein could be ascribed to the adsorption of AtTRB2 protein in GO that resulted in the reduction of the comparative distance between the active fluorescence emitter of protein and the quenching agent GO. Nevertheless, the fluorescence emission intensity variation suggested that the local environment of the chromophore residues changes after the addition of GO to the protein. It also reveals that the chromophore residues of bound protein were close to the GO nanosheet surface, which can result in the change of protein conformational due to a stronger association of protein and GO nanosheets.

**Figure 6:**
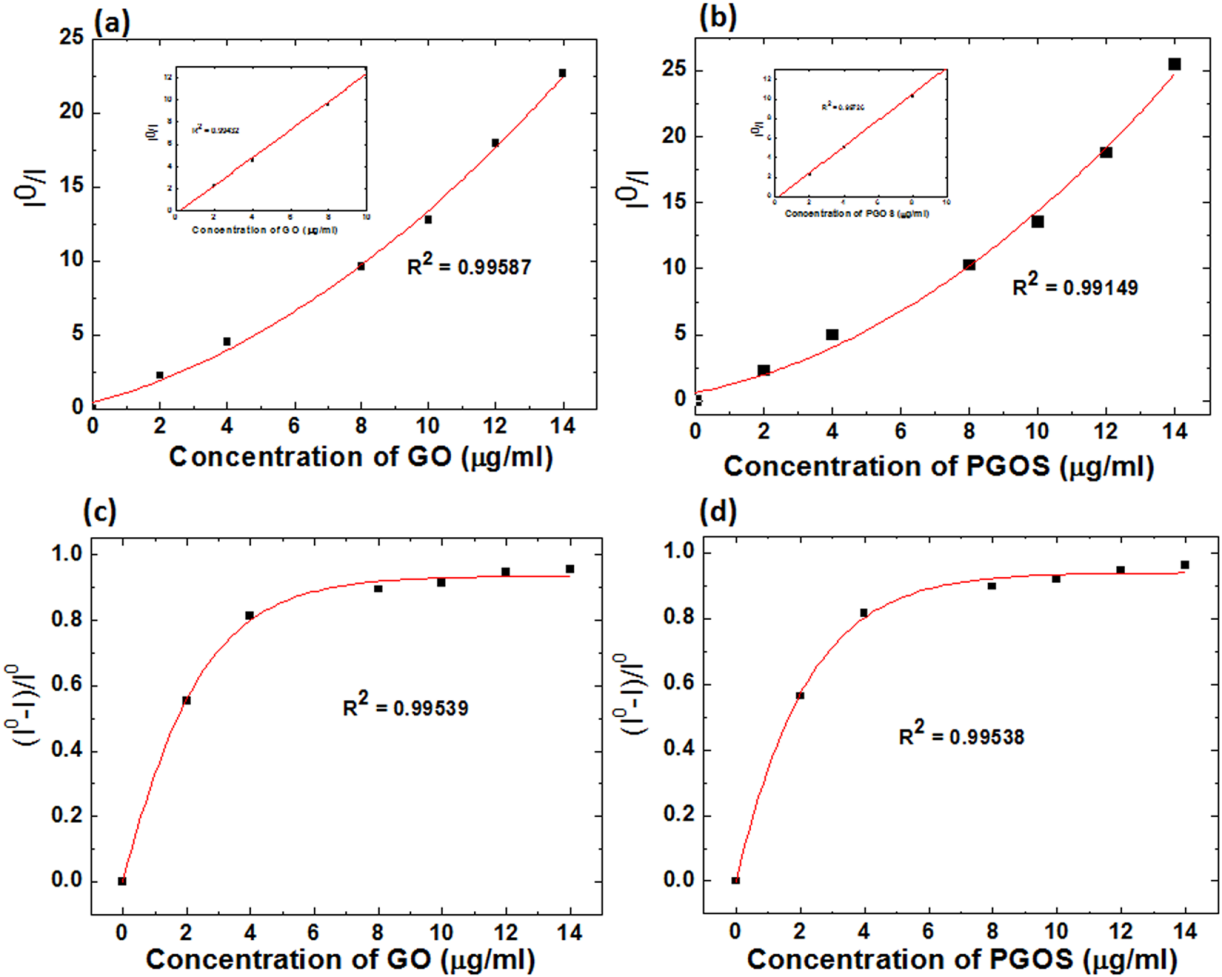
(**a**) Fluorescence quenching efficiency of AtTRB2 protein after treatment with GO at different concentration and (**b**) Fluorescence quenching efficiency of AtTRB2 protein after treatment with PGOS at different concentration; (**c**) Hill plots of the fluorescence quenching using GO at different concentration and (**d**) Hill plots of the fluorescence quenching using PGOS at different concentration.

To determine the nature of the fluorescence quenching, we then plotted a graph between the ratio of fluorescence emission intensity (I^0^/I) and GO or PGOS concentration, in which I^0^ and I represent the maximum emission intensity of protein in the absence and presence of GO or PGOS concentration, respectively, as shown in Fig. 6a and b. Figure 6 shows that I^0^/I exhibited an exponential trend with the increase in GO or PGOS concentration. This exponential trend implies that protein experienced both static and dynamic quenching in the presence of GO and PGOS. On the other hand, fluorescence quenching is primarily driven by diffusive transport at low GO or PGOS concentrations, as shown in inset Fig. 6. In both static and dynamic quenching, the following relation holds:

1 $${{\rm{I}}}^{0}/{\rm{I}}=(1+{{\rm{K}}}_{{\rm{SV}}}[{\rm{GO}}\,{\rm{or}}\,{\rm{PGOS}}])\,(1+{{\rm{K}}}_{{\rm{a}}}[{\rm{GO}}\,{\rm{or}}\,{\rm{PGOS}}])$$

where K_SV_ is the Stern-Volmer quenching constant, [GO or PGOS] is the concentration of the GO or PGOS nanosheets, and *K*_*a*_ is the association constant of the complex. Through this mathematical model, we can anticipate the fluorescence quenching efficiency (K_SV_) and K_a_ are the association constants of the complex (complex of protein with GO or PGOS). Using the non-linear regression method, we obtained the K_SV_ values of 0.26602 ± 0.06 and 0.27830 ± 0.02 (ml/µg) for GO and PGOS, respectively. The values of K_a_ were 0.26600 ± 0.08 and 0.27831 ± 0.03 (ml/µg) for GO and PGOS, respectively. This shows that K_SV_ and K_a_ values are slightly higher for PGOS than for the GO alone. However, the values do not significantly differ between GO and PGOS for quenching the AtTRB2 protein; hence, they have almost the same fluorescence quenching efficiency and association constant as those of the complex.

**Figure 7:**
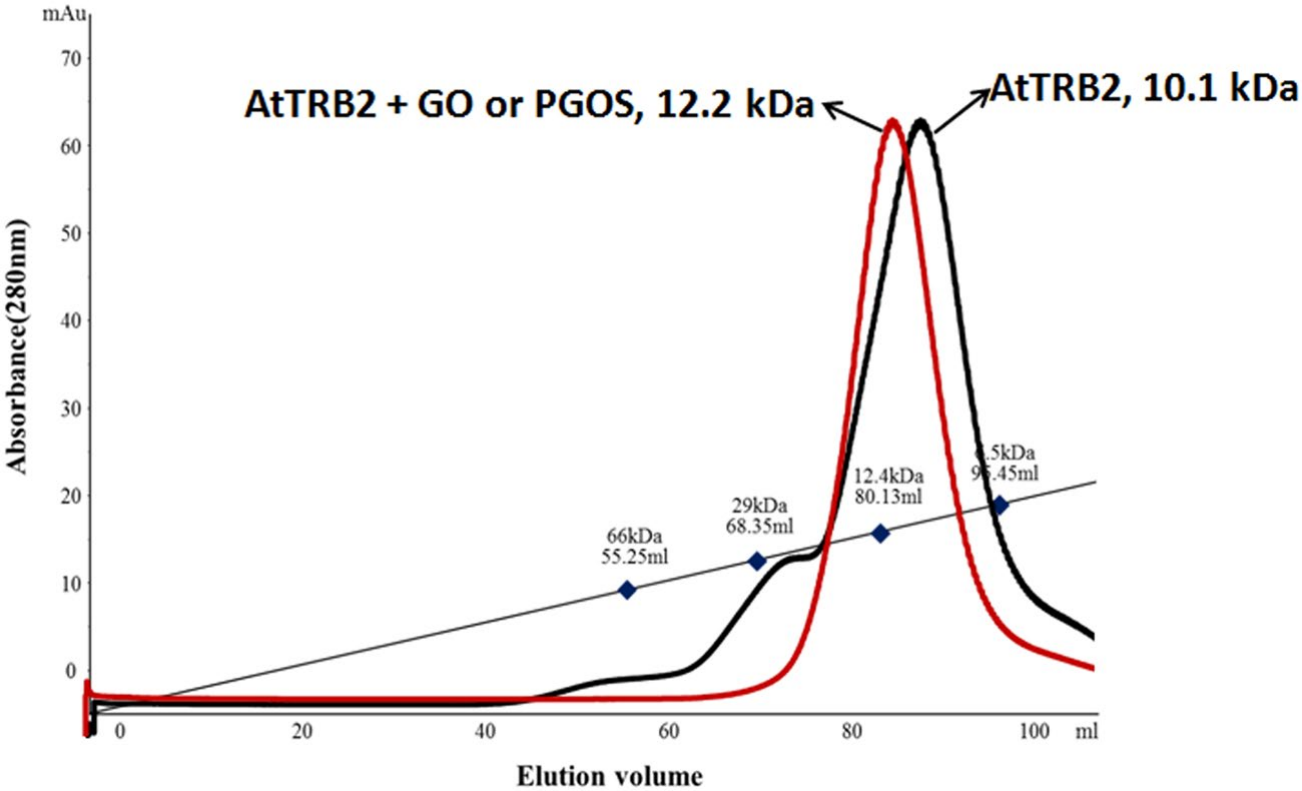
Size-exclusion chromatography (SEC) binding assay of AtTRB2 and GO.

To evaluate the binding dissociation constant (K_D_) and the Hill coefficient (n), we assumed that the binding of the AtTRB2 and GO or PGOS nanosheets occurred under equilibrium condition. By the non-linear curve fitting of the fluorescence quenching between AtTRB2 and GO or PGOS, we can derive the Hill equation, using Eqs 2 and 3

2 $$\frac{A}{{A}_{max}}=\,\frac{({I}^{0}-I)}{I}$$

3 $$\frac{A}{{A}_{max}}=\frac{{[GOorPGOS]}^{n}}{{{K}_{D}}^{n}+\,{[GOorPGOS]}^{n}}$$

where A_max_ is the saturation value of A, K_D_ is the equilibrium binding dissociation constant and n represents the Hill coefficient. K_D_ and n refer to the relative strength and cooperativity of the GO-protein interaction, respectively. From Fig. 6c, we observed that the K_D_ value was 1.6 ± 0.03 (µg/ml) and the Hill coefficient was 0.96 ± 0.07 for the GO-AtTRB2 complex, while the K_D_ and Hill coefficient for the PGOS-AtTRB2 complex was 1.5 ± 0.03 (µg/ml) and 0.95 ± 0.08, respectively, as illustrated in Fig. 6d. The Hill coefficient < 1 resembles the negatively cooperative reaction, which means the binding strength between AtTRB2 and the surface of GO or PGOS weakens gradually as the number of proteins adsorbed onto the GO or PGOS surface increases. Our experimental results show that the anti-cooperativity exhibited by the GO or PGOS-AtTRB2 protein complex could indicate changes in the physicochemical properties of the GO or PGOS nanosheets with the continuous adsorption of protein. This could be due to the diminution of the ionic or electrostatic binding energy of the GO or PGOS-AtTRB2 protein complex. The anti-cooperativity of the GO or PGOS-AtTRB2 protein complex reveals that AtTRB2 does not provide multiple binding sites to the GO nano-material; it also supports the above data, indicating that the interaction ability of GO and PGOS with AtTRB2 protein is almost same. As has been demonstrated, GO and PGOS do not show a significant difference in binding with AtTRB2 protein through the CD and fluorescence analysis; thus, in a further study we will focus on the GO binding. Moreover, we studied the size-exclusion chromatography (SEC) binding assay of AtTRB2 and GO. The experimental result shows that the size of AtTRB2 + GO (2 µg/ml) was 12.2 kDa, while the size was 10.1 kDa without GO, as shown in Fig. 7. This provides further evidence of the interaction of the GO and AtTRB2 protein. A further increase in the concentration of GO did not result in a further increase of size in the SEC assay, which also supports the negative cooperative reaction between GO and AtTRB2 protein. Additionally, the same results were observed for PGOS and AtTRB2 (data not shown). Both GO and PGOS bind to protein in similar ways, which reveals that RONS generated in PGOS has no role in the interaction. Hence, in later studies, we will investigate the GO with AtTRB2 to understand in more detail the specific amino acids that participate in binding with GO using ^1^H-^15^N NMR and docking simulation.

### ^1^H-^15^N NMR and docking simulation

**Figure 8:**
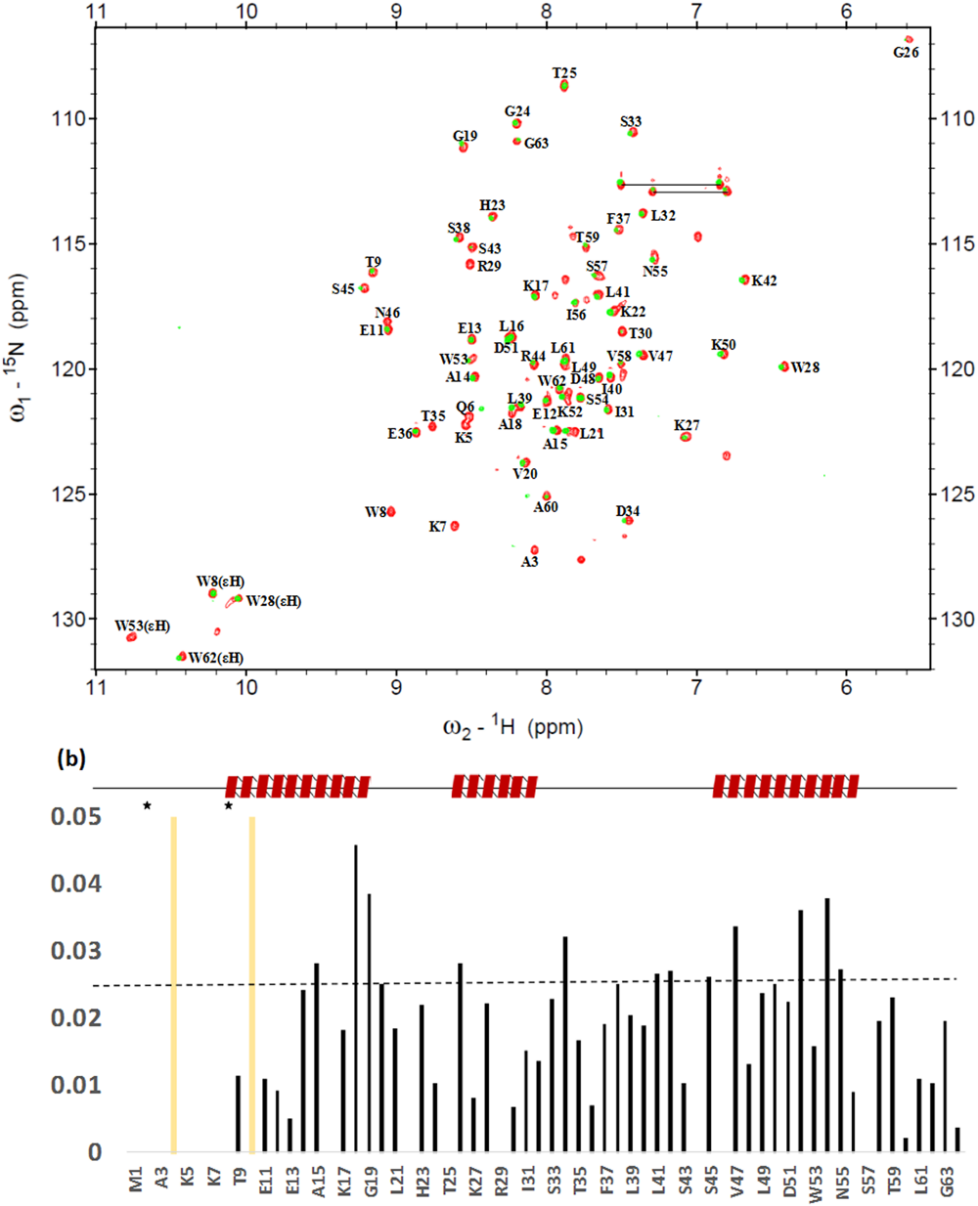
(**a**) ^1^H-^15^N HSQC spectra of AtTRB2 with or without treatment with GO (2 µg/ml). Native peaks of AtTRB2 in red color and GO-AtTRB2 in green color. Each peak corresponds to an amide N and H of each amino acid residue. (**b**) Amide chemical shift perturbation of AtTRB2 upon binding with GO.

The ^1^H–^15^N HSQC experiment confirms the formation of the AtTRB2 protein, as shown in our earlier work^47^. The resonance peaks shown in Fig. 8a are well-spread, indicating a well-defined tertiary structure^47^. In order to calculate the 3D structure, the 1000 upper distance limits were derived, and 45 dihedral angle constraints and 42 backbone hydrogen bond constraints were applied with the final 20 energy-minimized structures detected with van der Waals constraints of greater than 0.2 Å or angle restraints of greater than 5.0^0^ ^47^. The secondary structure of AtTRB2 contains three α-helices and connecting loops with a Myb domain. For these residues to maintain the secondary structure, the average root-mean-square deviation (RMSD) values relative to the mean coordinates of 20 representative conformers were calculated to be 0.41 ± 0.08 Å for the backbone atoms and 1.03 ± 0.13 Å for the heavy atoms. The final structures show no steric collisions between atoms, and thermodynamically fold into well-defined structures. The Pro10 to His23 residues for helix 1, the Trp28 to Ser33 residues for helix 2, and the Asn46 to Val58 residues for helix 3 belong to three α-helices in protein. The hydrophobic core consists of Leu16, Val20, Trp28, Ile31, Leu32, Leu41, Leu49, Trp53, and Ile56 residues, which help in stabilizing the overall tertiary structure of protein, as shown in Fig. 8a. The assigned details of the chemical shifts values and the atomic coordinates for the solution of AtTRB2 were described in earlier work^47^. After the treatment with the GO (2 µg/ml), we studied the ^1^H–^15^N HSQC and compared with the native protein (without GO treatment). Figure 8b shows that after the treatment of the AtTRB2 protein with GO, the K5, W8, E12, A15, A18, L21, G24, K27, T30, S33, E36, L39, K42, S45 D48, D51, R54, S57, A60, and G68 amino acids peak show a chemical shift, revealing that these amino acids interacted with GO nanosheets, resulting in quenching and structural changes. Further, to boost our NMR experiment and provide correlation between the experimental and theoretical study, we used docking simulation of GO and AtTRB2 protein.

**Figure 9:**
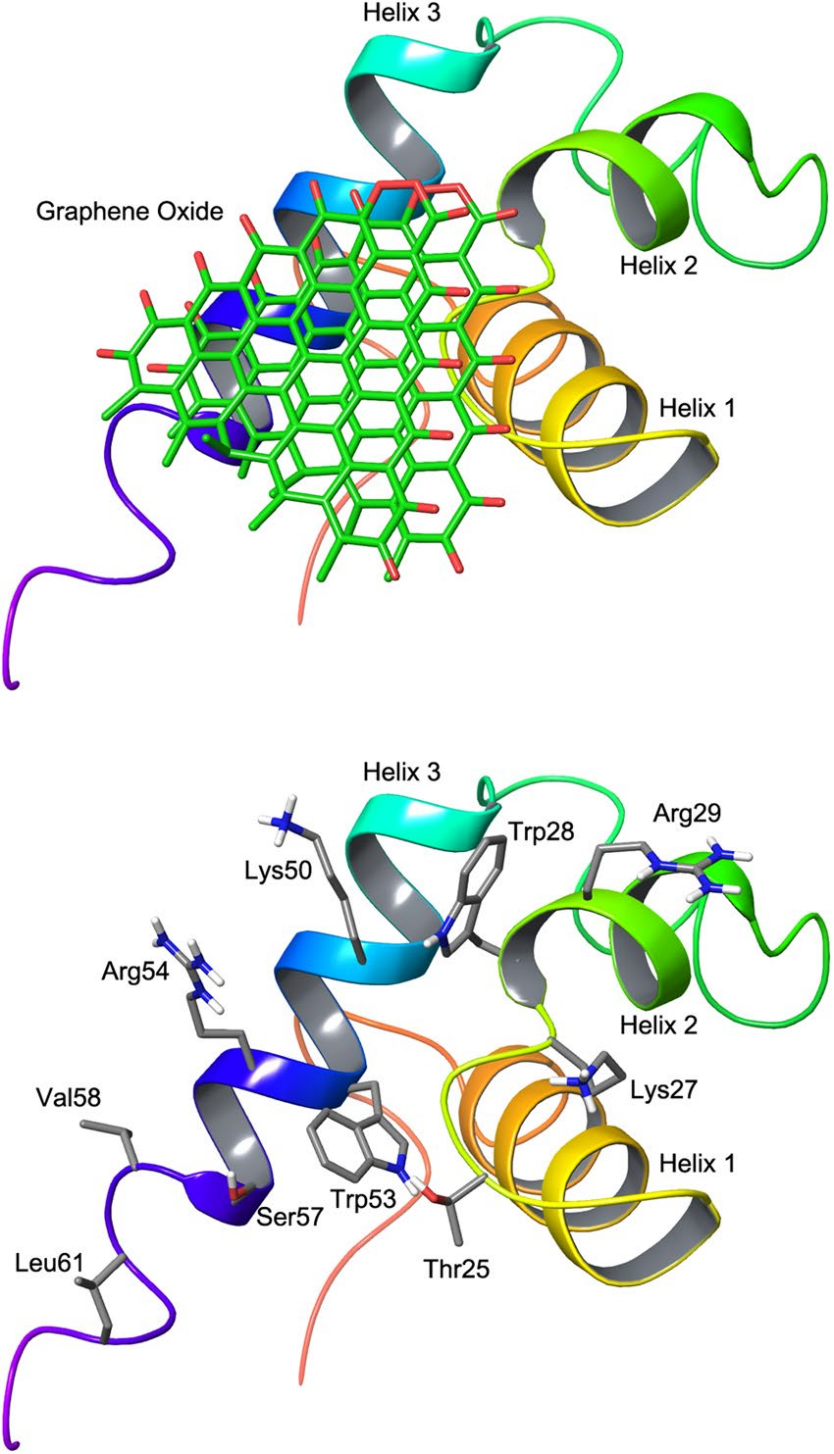
Predicted structure of telomere binding domain of AtTRB2 in complex with double-layer Graphene oxide (**a**) Binding pose of GO and (**b**) Binding site residues (within 5 Å of bound GO).

In order to observe how the GO sheet binds to the telomere binding domain of AtTRB2, we performed docking simulation, as shown in Fig. 9. Based on the docking result, the GO sheet was bound to the hydrophobic core of the binding domain which consists of the 2^nd^ and 3^rd^ helices and the loop between the 1^st^ helix and 2^nd^ helix. The binding energy of GO was −11.2 kcal/mol. The residues involved in direct interaction (<3 Å) with the GO sheet were Gly26, Lys27, Trp28, Trp53, Arg54, Ser57, and Leu61. This was consistent with the NMR data, which indicated our experimental results are very closely correlated with the docking simulation.

Hence, GO has the ability to bind to the telomere binding protein, so the probable mechanism of the cancer cell growth cessation.

## Conclusion

Although, the treatment of the GO and f-MWCNT solutions with atmospheric pressure plasma does not alter the structure of GO and f-MWCNT, but it resulted in generation of the reactive species into the solution. Additionally, no significant difference in the decrease concentration of hTERT after treatment with GO and PGOS. This attributes to the fact that RONS generated in PGOS does not contribute to the decrease of hTERT concentration considerably. Also, f-MWCNT and PCNTS were unable to decrease the hTERT concentration. Latter, we investigated the structural distortion and protein binding ability of GO and PGOS using AtTBR2 (telomere binding protein). The structural distortion was studied using the CD and HSQC NMR spectroscopy, whereas the binding ability was studied using fluorescence spectroscopy and SEC binding assay. The CD and fluorescence results revealed that both GO and PGOS have the ability to bind to AtTBR2 protein, but their binding abilities were not considerably different from each other. The ^1^H-^15^N HSQC and docking simulation experiments showed that the GO sheet was bound to the hydrophobic core of the binding domain, consisting of the 2^nd^ and 3^rd^ helices and a loop between the 1^st^ and 2^nd^ helix. It is anticipated that this study will increase the future utilization of biomaterials for treatment of cancer.

## Electronic supplementary material

Supporting information
