# Supplementary material for: Interaction studies of carbon nanomaterials and plasma activated carbon nanomaterials solution with telomere binding protein
Source: Sci Rep. 2017 Jun 1;7:2636. doi: 10.1038/s41598-017-02690-4 (PMC5454022; doi:10.1038/s41598-017-02690-4)
Supplement: Supplementary file 1 — Supporting information [file 41598_2017_2690_MOESM1_ESM.pdf]

Supporting file

Interaction studies of carbon nanomaterials and plasma activated carbon nanomaterials solution with telomere binding protein

Pankaj Attri*[1], Jitender Gaur‡[2], Sooho Choi‡[3], Minsup Kim[4], Rohit Bhatia[2], Naresh Kumar[1], Ji Hoon Park[1], Art. E. Cho[4], Eun Ha Choi*[1] and Weontae Lee*[3]

[1]*Plasma Bioscience Research Center/Department of Electrical and Biological Physics, Kwangwoon University, Seoul, Korea 139–701*
[2]*Division of Sustainable Technology, Rudraksh Prodhyogiki Sangathan. Delhi, India.*
[3]*Department of Biochemistry, College of Life Science & Biotechnology, Yonsei University, Seoul 120–749, Korea*
[4]*Department of Bioinformatics, Korea University, Sejong 02841, Korea.*

Figure Captions

Fig. S1: Standarization process of the size exclusion chromatography (a) Molecular weight of standard protein and (b) Determination of molecular weight of AtTRB2 using equation, log Y = -1.2483x + 6.2868, R^2 = 0.9943, x = elution volume.

Fig. S2: (a) XPS C(1s) spectra of thin GO sheets; (b) Raman spectra, blue- thin GO sheets and black- CNH; (c) SEM image of GO sheets synthesized by unzipping of CNHs; (d) Electron diffraction (thin GO sheets) spots are labeled with Miller-Bravais indices.

Fig. S3: Secondary structure ofAtTRB2 protein with different concentation of GO (black) and PGOS (red) (a) 2 µg/ml; (b) 4 µg/ml and (c) 8 µg/ml.

Fig. S4: Secondary structure ofAtTRB2 protein with different concentation of GO (black) and PGOS (red) (a) 10 µg/ml; (b) 12 µg/ml and (c) 14 µg/ml.

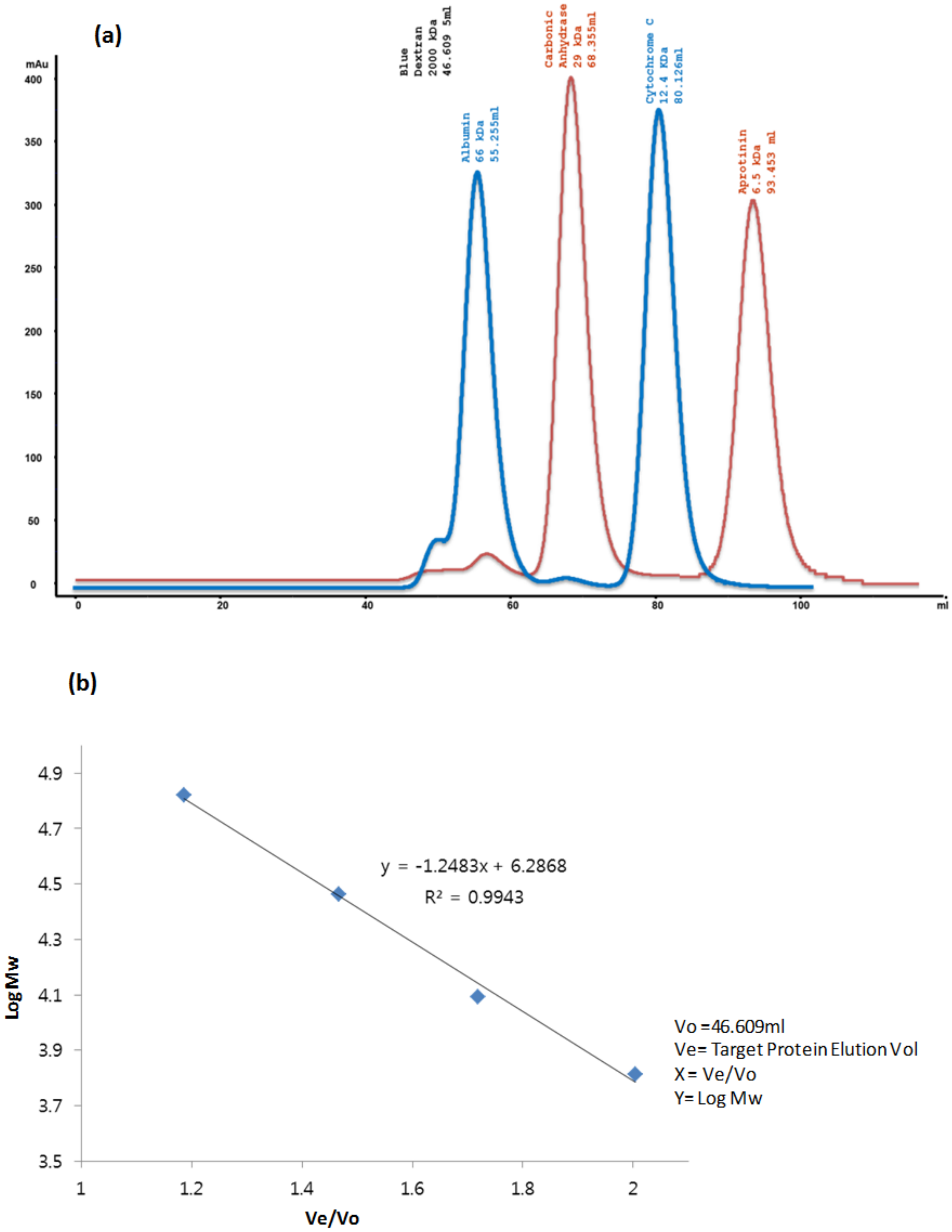
(a)
mAu
400
350
300
250
200
150
100
50
0
0
20
40
60
80
100
ml
Blue Dextran 2000 kDa 46.609 5ml
Albumin 66 kDa 55.255ml
Carbonic Anhydrase 29 kDa 68.355ml
Cytochrome C 12.4 KDa 80.126ml
Aprotinin 6.5 kDa 93.453 ml
(b)
4.9
4.7
4.5
4.3
4.1
3.9
3.7
3.5
Log Mw
1
1.2
1.4
1.6
1.8
2
Ve/Vo
y = -1.2483x + 6.2868
R² = 0.9943
Vo =46.609ml
Ve= Target Protein Elution Vol
X= Ve/Vo
Y= Log Mw

Fig. S1

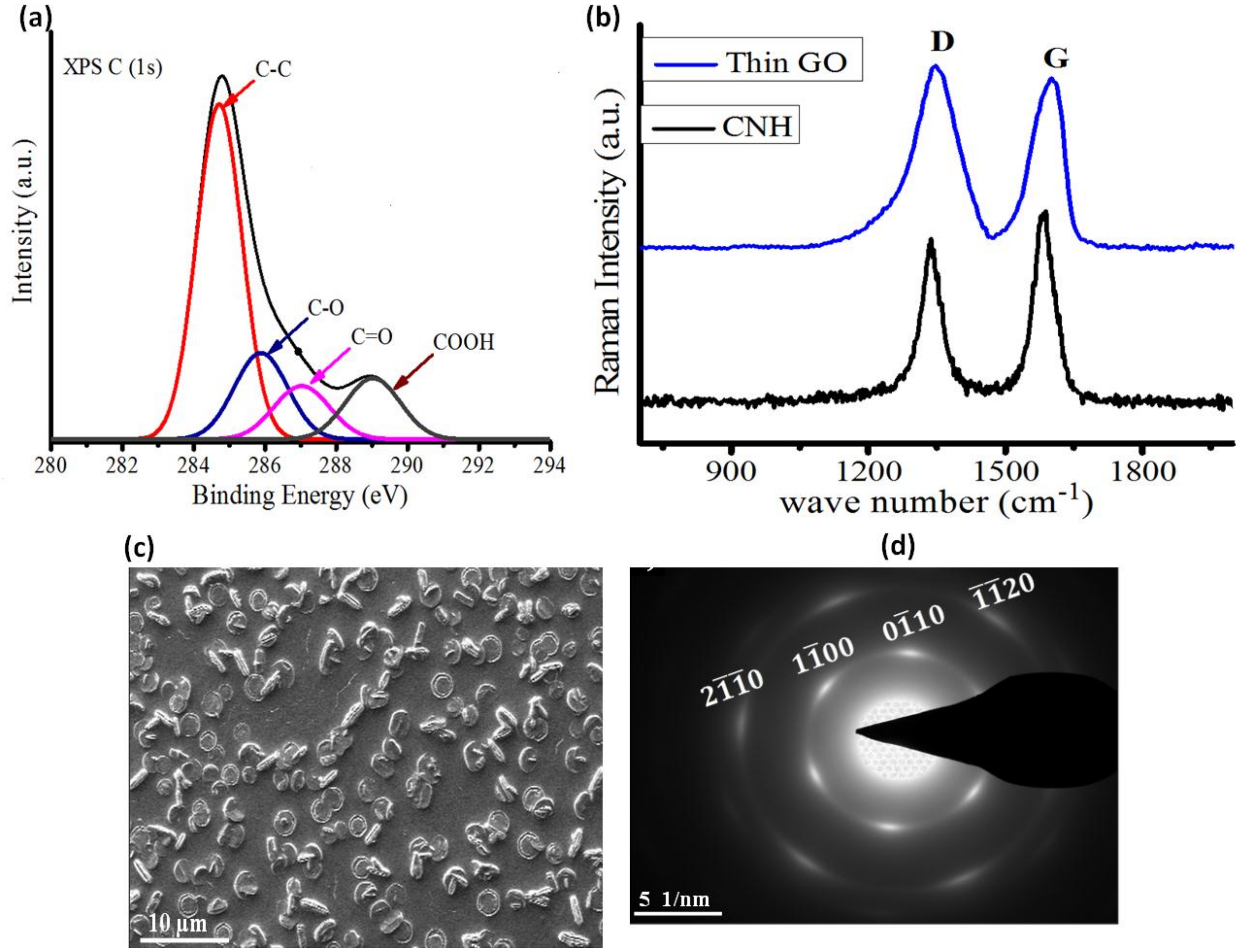
(a)
XPS C (1s)
C-C
C-O
C=O
COOH
Intensity (a.u.)
280 282 284 286 288 290 292 294
Binding Energy (eV)
(b)
Thin GO
CNH
D
G
Raman Intensity (a.u.)
900 1200 1500 1800
wave number (cm^{-1})
(c)
10 µm
(d)
$2\bar{1}\bar{1}0$
$1\bar{1}00$
$0\bar{1}10$
$\bar{1}\bar{1}20$
5 1/nm

Fig. S2

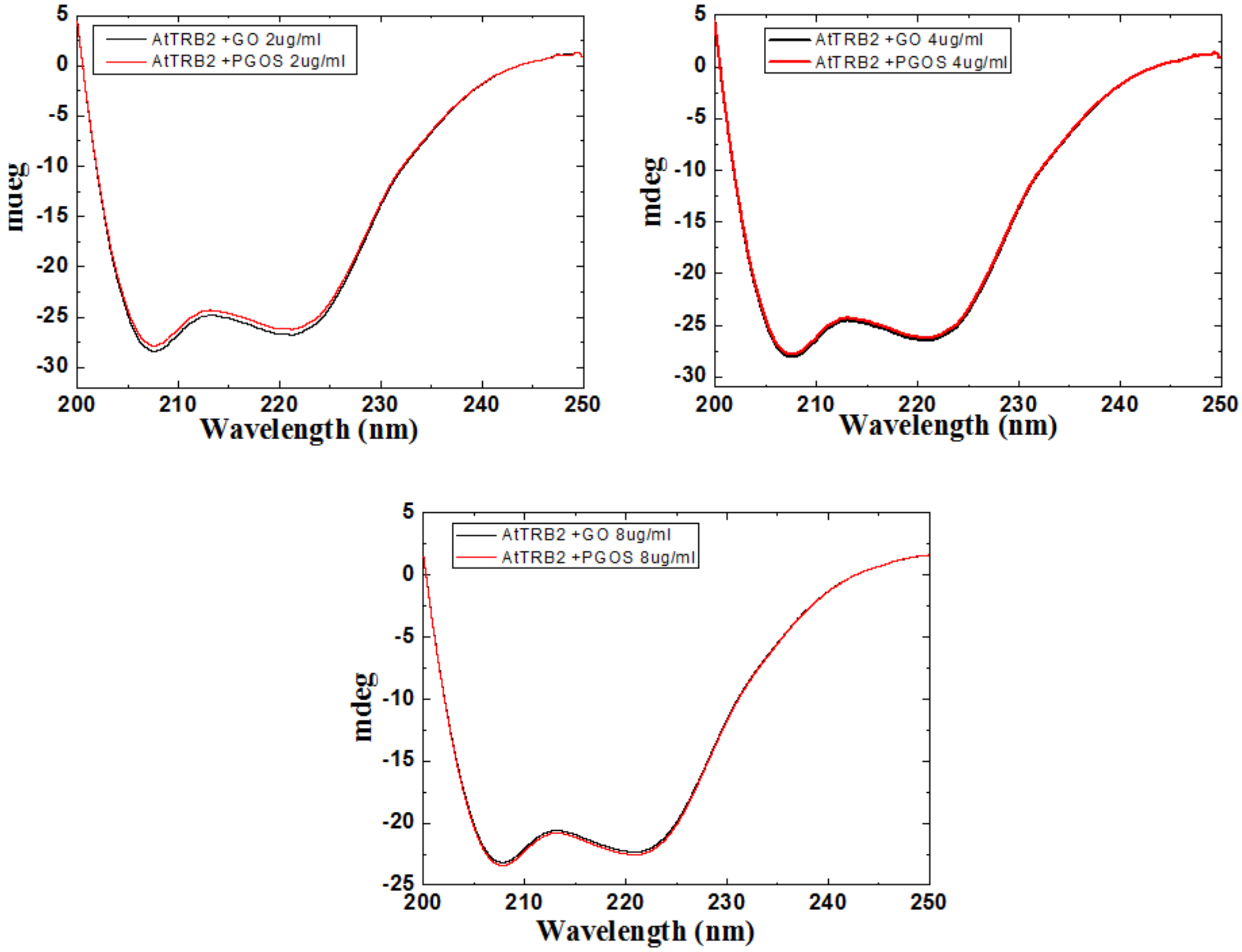
AtTRB2 +GO 2ug/ml
AtTRB2 +PGOS 2ug/ml
mdeg
Wavelength (nm)
AtTRB2 +GO 4ug/ml
AtTRB2 +PGOS 4ug/ml
mdeg
Wavelength (nm)
AtTRB2 +GO 8ug/ml
AtTRB2 +PGOS 8ug/ml
mdeg
Wavelength (nm)

Fig. S3

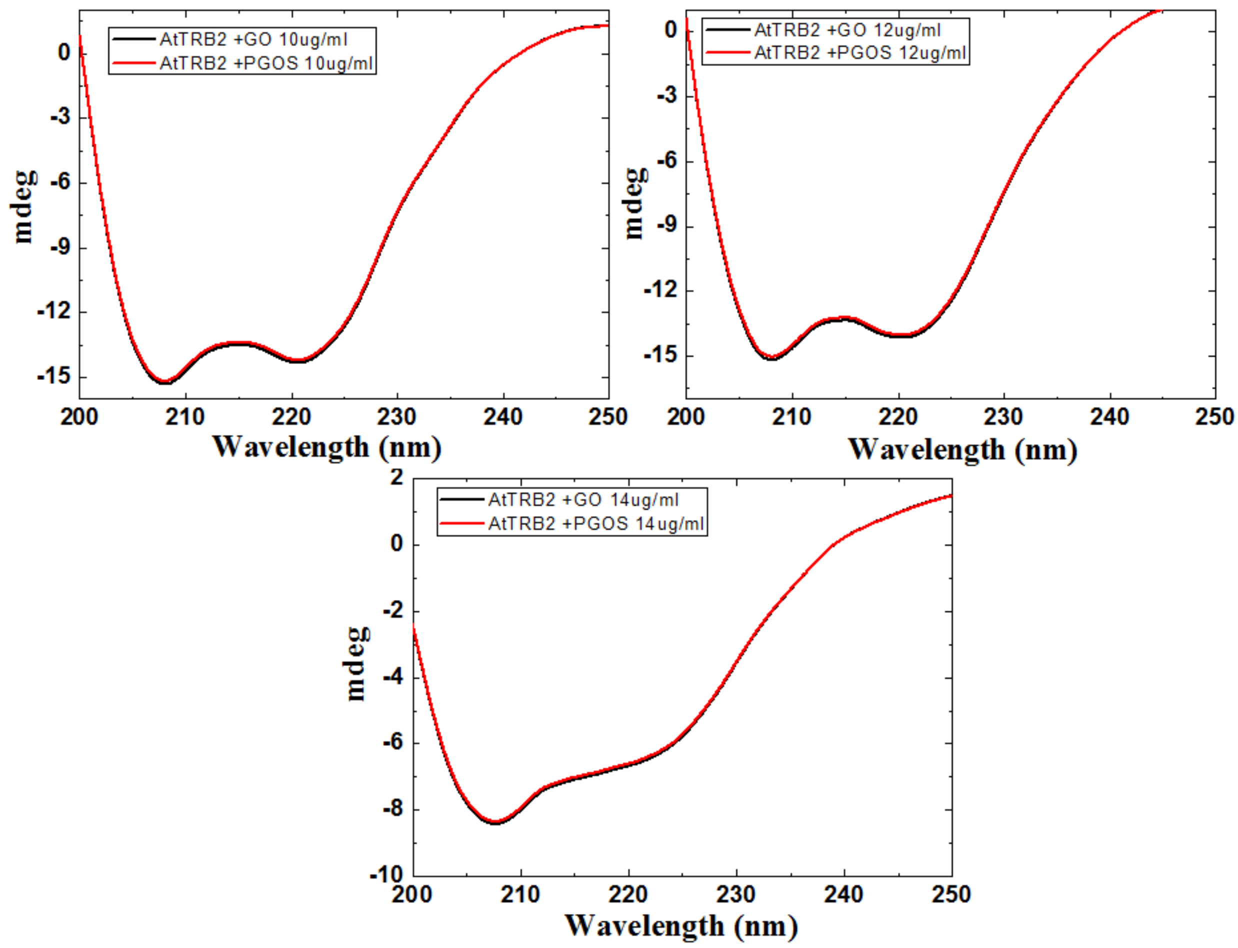
AtTRB2 +GO 10ug/ml
AtTRB2 +PGOS 10ug/ml
mdeg
Wavelength (nm)
AtTRB2 +GO 12ug/ml
AtTRB2 +PGOS 12ug/ml
mdeg
Wavelength (nm)
AtTRB2 +GO 14ug/ml
AtTRB2 +PGOS 14ug/ml
mdeg
Wavelength (nm)

Fig. S4
